# Preparing Genetic Counselors for Advocacy Partnerships: A Novel Internship Model

**DOI:** 10.1002/jgc4.70289

**Published:** 2026-09-15

**Authors:** Marin Kalista, Lisa Jay Kessler, Deborah Requesens, Kathleen D. Valverde, Rebecca Mueller

**Affiliations:** ^1^ Master of Science in Genetic Counseling Program University of Pennsylvania Perelman School of Medicine Philadelphia Pennsylvania USA; ^2^ Orphan Disease Center JumpStart Program University of Pennsylvania Philadelphia Pennsylvania USA

**Keywords:** disability, genetic counseling education, internship, lived experience, patient advocacy, patient advocacy organizations, professional identity

## Abstract

Patient advocacy organizations (PAOs) and genetic counseling have expanded in tandem with the growth of precision medicine. Genetic counseling and PAOs share important goals related to advancing genomic medicine, creating novel possibilities for synergistic collaboration, especially as the sheer number of PAOs focused on genetic conditions presents new options for genetic counselor education. While patient advocacy has long been part of genetic counseling education, there are few published reports or studies focused on how advocacy is incorporated into genetic counseling graduate training. Accordingly, the University of Pennsylvania Master of Science in Genetic Counseling Program and the University of Pennsylvania Orphan Disease Center's JumpStart Program collaborated to launch an experiential service‐learning opportunity wherein genetic counseling trainees partner with a disease‐specific advocacy group over the course of a 10‐week advocacy internship. Here, we describe our evolving efforts to incorporate an advocacy internship in our graduate curriculum, culminating in the development of The Genetic Counseling Student Exchange (GCSX) program. We share the curricular format, planning and staff workflow, as well as examples of internship project deliverables. We also reflect on the internship from varying perspectives, highlighting our experiences and feedback from different stakeholders. In closing, we consider how the GCSX may be a key step towards incorporating more disability education into our genetic counseling training program while calling for future research on GCSX to measure its impact and to determine how best to tailor disability education around the internship experience. Overall, we hope that detailing the GCSX inspires other GC programs to consider adapting aspects of the educational framework that we have developed to expand advocacy training across genetic counseling programs.

## Plain Language Summary

Genetic counseling educational standards include patient advocacy, but there are few published accounts of how genetic counseling programs incorporate advocacy into coursework. In this paper, we describe a patient advocacy internship developed at the University of Pennsylvania and reflect on our four years of coordinating the internship program.

**What is Known About This Topic:**

As their respective roles grow in the era of precision medicine, genetic counselors (GCs) and patient advocacy organizations (PAOs) increasingly engage as partners. Advocacy has long been a part of genetic counseling training, yet little is known about how programs incorporate advocacy into curricula.

**What the Paper Adds to the Topic:**

Here, we present a novel model for incorporating patient advocacy into genetic counseling training by describing our Genetic Counseling Student Exchange Program with patient advocacy organizations.

## 1 Introduction

Over the past two decades, patient advocacy organizations have become vital collaborators in rare disease research. Whereas patient advocacy organizations (PAOs) were historically understood as allies for raising awareness and connecting patients with support networks (Weiss 1992), they are now recognized as essential partners in shaping research priorities, influencing clinical trial development, and ensuring equitable participation in science and healthcare (Koay and Sharp 2013; Merkel et al. 2016; Nori et al. 2022; Patterson et al. 2023). As PAOs have grown, so too has the genetic counseling profession as both have expanded in tandem with genomic research, the discovery of novel rare conditions, and the rapid development of targeted therapies (Baty 2018; Dunkle 2014; Field et al. 2016; Muraresku et al. 2024; Ormond et al. 2018), presenting important opportunities for synergistic collaboration.

Within this context, GCs are increasingly involved with PAOs in a variety of roles (Madden 2025). A recent survey study indicates that ~65% of the sampled disease‐specific advocacy organizations have a working relationship with a genetic counselor, ranging from volunteer work to full‐time employment, with GCs doing variant interpretation, serving on panels and advisory committees, or presenting as conference speakers for organizations (Roulston et al. 2024). Review of National Society of Genetic Counselors (NSGC) Professional Status Surveys (PSS) appears to corroborate this trend, indicating that as of 2024, 7% of GCs report engaging in advocacy as a part of their roles, while 12% of self‐employed or consulting GCs contract with advocacy organizations (National Society of Genetic Counselors 2024).

The connection between advocacy organizations and the genetic counseling profession is reflected in accreditation requirements and graduate admission processes. The Accreditation Council for Genetic Counseling (ACGC) standards require genetic counseling master's programs to provide students with opportunities to engage with support groups and other advocacy organizations as a relevant experience to supplement fieldwork education (Accreditation Council for Genetic Counseling 2023b). When applying to genetic counseling graduate programs, applicants are encouraged to list their volunteer experiences with patient support groups and advocacy organizations.

Despite the profession's apparent prioritization of collaborating with advocacy organizations, little has been written about the role of contemporary advocacy in genetic counseling graduate training. Historical research demonstrates that early in the 1980s and 1990s, several genetic counseling programs prioritized community‐based training (Hogan 2022; Stern 2012). This type of immersion, which took place at schools including Brandeis University, was meant to facilitate engagement with community advocacy through experiential learning focused on disability. Since then, programs have incorporated advocacy into curricula in a variety of ways, but these efforts are primarily evident in course rosters, social media posts, and genetic counseling program websites rather than programmatic reports or empirical research.

At the University of Pennsylvania (Penn), we have developed a formal advocacy internship to address the expanding collaboration between GCs and PAOs. The advocacy internship, termed the Genetic Counseling Student Exchange (GCSX), is a fieldwork placement integrated into the Penn Master of Science in Genetic Counseling (MSGC) Program. It was designed and coordinated through collaboration between the Penn MSGC's fieldwork director and associate director (LJK) and the director of a University of Pennsylvania program called JumpStart (DR). Jumpstart is a program housed with the Orphan Disease Center at the University of Pennsylvania and the Children's Hospital of Philadelphia (CHOP), that serves to establish and progress research agendas in emerging and neglected rare diseases by working closely with key stakeholder groups. The advocacy internship allows students to gain exposure to the work of PAOs, to make connections with leaders of advocacy organizations, and to identify opportunities to share resources and collaborate. The purpose of this paper is to offer ideas and a framework for future genetic counseling students and programs to develop training opportunities focused on patient advocacy within their institutions. To achieve this, we narrate our evolving efforts to develop and grow the advocacy internship, sharing the curricular format, planning, and staff workflow. We also present select examples of internship project outcomes and describe reflections on the internship experience from varying perspectives. In closing, we reflect on our pedagogical goals and anecdotal observations in developing and operating the advocacy internship, offer additional ideas for curricular innovation, and identify gaps in our knowledge of the program's impact to articulate a need for future research to rigorously evaluate the GCSX program. Overall, we hope to inspire other genetic counseling programs to explore options for expanding patient advocacy training within genetic counseling graduate education.

## 2 Our Positionalities as Advocates, Learners, and Educators

As a group of educators (LJK, DR, RM, KDV) and a current genetic counseling trainee (MK), we bring a variety of perspectives to our reflections on the advocacy internship presented here. We have educational backgrounds in disability studies (RM, LJK) and rare disease advocacy (MK) and we are advocates for disability inclusivity and education within genetic counseling. Our positionalities include dominant and marginalized identities, including having a disability (RM), being the parent of a child with a disability (KDV), and Hispanic heritage (DR), which attune us to social barriers to societal inclusion and geographic, linguistic, and economic barriers to inclusion in biomedical research and advocacy, respectively. We work closely with organizations focused on advancing therapeutics for rare diseases (DR, RM) and all have both personal and professional connections with advocacy organizations. With respect to the advocacy internship, we are also all guided by LJK's professional mission to promote an inclusive and flexible professional identity within genetic counseling and DR's commitment to advancing therapeutic development for rare diseases through collaboration among patient organizations, academia, and industry. It is through these varied lenses and commitments that we both built and reflect upon the advocacy internship.

## 3 Advocacy Internship Development

### 3.1 Early Piloting of an Advocacy Internship

We launched our first advocacy internship at Arcadia University in 2012, before our genetic counseling program moved to Penn in 2019. The advocacy internship was inspired by LJK's observation that GCs were increasingly playing leading roles in PAOs. The inaugural advocacy internship at Arcadia involved one student and one foundation, the Friedreich Ataxia Research Alliance (FARA) led by GC Jennifer Farmer, an alumna, friend, and colleague of our GC program. At the time, LJK was a part‐time (0.5 FTE) fieldwork coordinator for Arcadia University. Coordinating the internship took LJK about twice as long as coordinating a typical fieldwork rotation, given the need to develop rotation objectives for a non‐clinical internship experience and familiarize a participating student with the different types of expectations and roles involved in rotating with FARA.

In subsequent years, additional students interned at FARA. This early version of the advocacy internship involved first‐year students who served 6 h per week for 6 weeks, often working onsite in the FARA office, where they joined a small team that ran the organization. As part of the experience, students supported FARA to prepare for a conference and received training to moderate a session with FARA members. Being integrated into the organizational workflow allowed students to learn the lingo and feel part of the team. Student interns were also required to interview a person with FA and write a reflection essay to further acquaint them with the lived experience of the condition.

Through internship evaluations, students shared that their time at FARA helped them to learn more about PAOs, giving them confidence to assess organizations and refer patients to similar groups in the future. The students who interned with FARA became the student leads for an annual volunteer experience each fall. One of the students was inspired to work with FARA for her master's thesis, so the experience with the team led to a separate research project. Several interns were offered summer positions with FARA assisting with clerical tasks, which benefited students as well as the organization. Several of our alums also went on to take positions working at FARA. Leveraging alumni contacts led to the FARA internship experience, master's thesis projects with the organization, and full‐time genetic counseling positions for graduates. The success of the advocacy internship at FARA motivated us to propose a similar partnership with Susan Dickinson, an alum serving as the Chief Executive Officer at the Association for Frontotemporal Dementia, which also began to host advocacy interns.

The work of students in this early iteration of the advocacy internship provided justification for the relevance of an advocacy experience to genetic counseling training; however, this pilot was not guided by a formal curricula structure nor was it available to every student enrolled in the genetic counseling program. As GC roles in PAOs continued to grow, LJK aimed to expand the advocacy internship in the hopes of eventually forging opportunities to place more students with different advocacy organizations.

### 3.2 Defining a Pedagogical Approach: Service‐Learning

In planning to expand and formalize genetic counseling training in patient advocacy, we employed a service‐learning model of education to ensure that the program was guided by the needs and priorities identified by the community organizations themselves. Service‐learning began in the undergraduate setting and was later incorporated in health profession education (Jacoby 1999; Seifer 1998). Service learning combines community service experience with structured learning objectives, preparation, and reflection, and it aims to benefit both the education of students and the communities served (Seifer 1998). Though service learning has been studied and documented within other areas of health profession education (Sabo et al. 2015; Stewart and Wubbena 2014), it has not been formally described within the genetic counseling literature. With service learning as the guiding pedagogical approach, we looked for partnerships to expand the advocacy initiative beyond just two foundations so that all 18 of our first‐year students could participate in an advocacy internship.

### 3.3 Expanding to the Genetic Counseling Student Exchange Program

In 2019, our genetic counseling program made an institutional transition from Arcadia University to Penn's School of Medicine. As we acclimated to Penn, we learned that we were uniquely positioned to launch a larger advocacy training initiative for genetic counseling students. At Penn, LJK's effort as fieldwork director and associate director became a full‐time (1.0 FTE) position, a small percentage of which is allocated to forging new collaborations to develop novel student learning experiences.

At Penn we joined forces with JumpStart, a program within the University of Pennsylvania's Orphan Disease Center where DR directs the JumpStart program full‐time. While DR's directorship of JumpStart extends far beyond connecting genetic counseling students and advocacy organizations, this work is a synergistic part of the program's overarching mission to establish and progress research agendas in emerging and neglected rare diseases. By working closely with patient groups, foundations, pharmaceutical and biotechnology companies, and the academic community, JumpStart strives to drive therapeutic development for rare diseases forward. The organization considers connecting rare disease groups with academics and learners to be a central part of their mission. In working closely and regularly with a wide range of PAOs, DR has developed a deep understanding of the types of needs that organizations have for volunteer work and support by different types of academics and learners. She ensured that any efforts to build or expand the advocacy rotation were guided by the evolving needs expressed by the leadership and staff of PAOs. The enthusiastic interest and support of PAOs was confirmed prior to building out the advocacy internship.

With a shared vision of connecting PAOs with genetic counseling student interns, JumpStart and the Penn MSGC program launched the Genetic Counseling Student Exchange (GCSX) program in 2019 to provide all 18 MSGC students with a formal advocacy internship in the second term of their first year of the graduate program. The work of launching the GCSX was divided between LJK in her capacity as fieldwork director and DR, the director of Jumpstart. Both spent approximately 40 h developing and launching the first GCSX internship program.

The second semester of the first year was chosen for the advocacy rotation because by that point students have gained baseline knowledge of genetic testing and medical terminology through an observational clinical rotation and coursework and have started a laboratory genetics rotation that further acquaints them with the complexity of genomic analysis and test reporting. Placing the advocacy internship after their observational rotation and before their first participatory rotation also aims to help students gain practice translating medical concepts into patient‐friendly language before they are counseling patients in clinic. Additionally, scheduling the advocacy rotation in the spring of the first year had logistical benefits for our MSGC program as it left clinical sites open for second‐year students completing their final participatory rotations.

## 4 The Genetic Counseling Student Exchange (GCSX) Advocacy Internship

### 4.1 Coordination and Set‐Up

**FIGURE 1:**
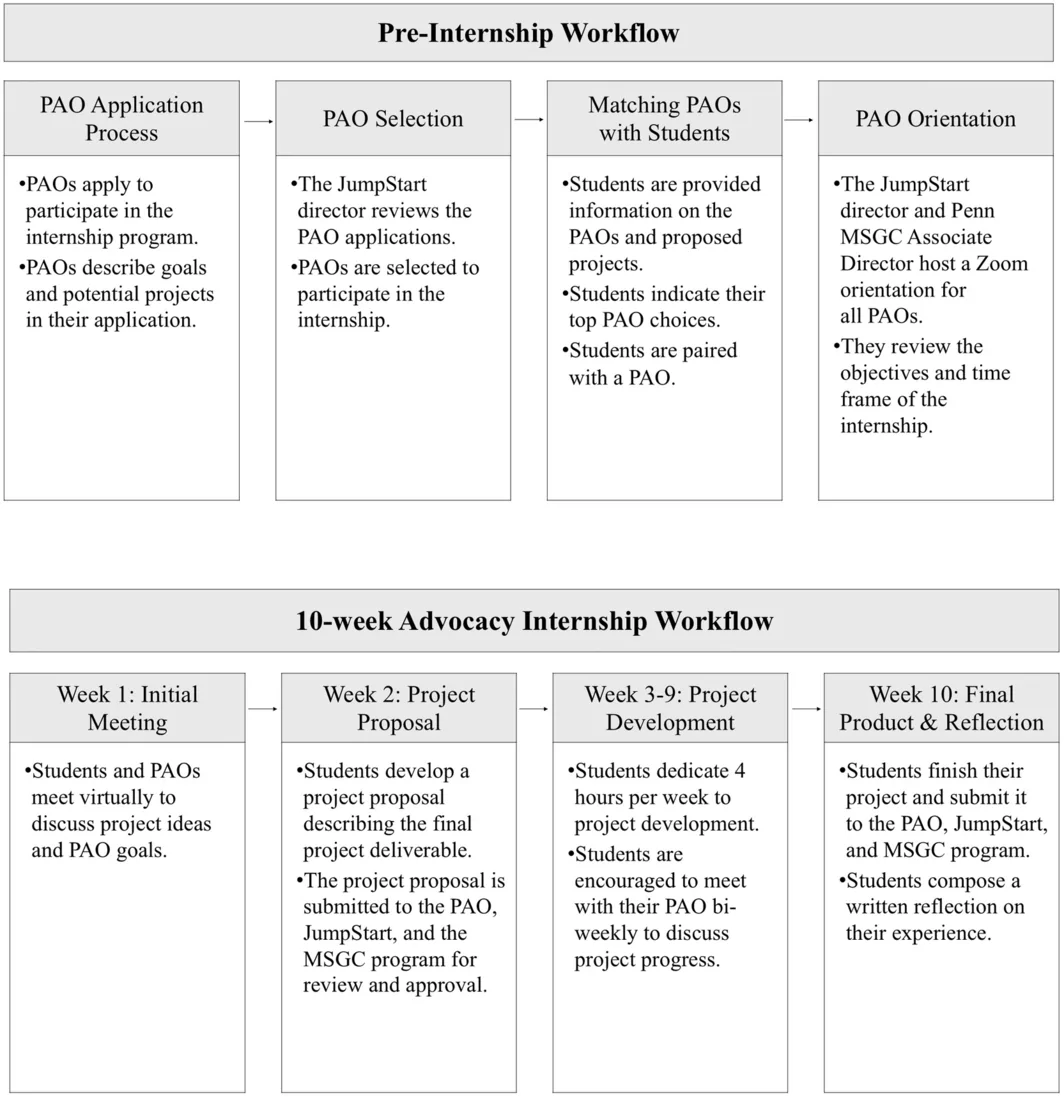
Workflow for recruiting and orienting patient advocacy organizations (PAOs) and matching students with PAOs for the internship followed by the 10‐week advocacy internship workflow.

Coordinating the GCSX advocacy internship now takes about half the time needed to coordinate clinical placements. DR solicits interest via a short application from rare disease PAOs that includes potential projects, as noted in Figure 1. This annual call for PAO advocacy intern sites and projects aims to ensure that the internship is responsive to the evolving priorities and needs of different advocacy organizations. DR then selects PAOs based on the feasibility of proposals given learners' skill level and expected time commitment. DR gives a presentation about JumpStart and PAOs to prepare students for their internships. LJK shares the placement opportunities with MSGC first‐year students and elicits their preference rankings to match learners with PAOs. Finally, LJK and DR run an hour‐long orientation for participating PAO leadership to establish the objectives and timeframe of the internship to promote equitable benchmarks for all learners.

### 4.2 Structure

The GCSX internship is a formal 10‐week experience in which students dedicate 40 h towards completion of an advocacy project. Project deliverables are tailored to the needs of each PAO; during the first week of the internship, learners meet with their PAO to elicit needs and to brainstorm possible project deliverables. The internship counts as fieldwork, and learning experiences are tracked in Typhon, the Student Tracking System for Higher Education. Students are required to complete the advocacy internship just like a clinical rotation; the full‐time MSGC program is designed such that first‐year internships, including the advocacy internship, are included within the block tuition. Work is typically conducted remotely in a self‐paced manner, while regular virtual meetings with PAO partners provide ongoing guidance and support throughout the project. Students are expected to dedicate approximately 4 h per week to their projects, and the self‐paced design of the internship ensures flexibility so that students can balance other school responsibilities.

Given the variability in PAO leadership, size, stage, and initiatives, as well as learners' skills and interests, advocacy internship experiences vary considerably. As with fieldwork experiences, we strive for equitability by defining the time commitment and workflow of the internship, as outlined in Figure 1, while allowing learners and PAOs to tailor each specific project. The fieldwork director (LJK) tracks learners' advocacy internship experiences by reviewing all completed internship projects and learners' reflective statements on a pass/fail basis.

Additionally, all learners have MSGC academic advisors who regularly check in about all internship and fieldwork experiences, ensuring continuous feedback on the advocacy internship while requiring little additional effort beyond standard academic advising. Learners' reflective statements and informal conversations about internships in courses and meetings also provide insight into the ways that learners have interacted with their PAOs. Collectively, reflections suggest that many, but not all, learners are consistently interacting with PAO leadership who are parents of affected children. As a result, conversations with PAO leadership typically involve learning about the lived experience of parenting a child with complex medical conditions and disabilities, which helps to augment and expand students' exposure beyond medical depictions of such conditions. Many of the project deliverables are patient‐friendly guidance and condition‐specific information that is developed via frequent interaction with PAO representatives who typically share personal and/or community‐informed perspectives on the experiences, priorities, and needs of key stakeholders.

### 4.3 Learning Goals

The GCSX advocacy internship aims to facilitate an immersive, hands‐on experience in rare disease advocacy and research. By framing the community‐based advocacy experience with discrete learning goals, as outlined in Table 1, and concluding the experience with a required written reflection, the GCSX advocacy internship mobilizes principles of service‐learning (Jacoby 1999; Seifer 1998) to equip learners with skills that align with ACGC's Practice Based Competencies (PBCs) (Accreditation Council for Genetic Counseling 2023a). The learning objectives of the GCSX internship address many of the PBCs, spanning competencies related to genomics knowledge and expertise, communication, research, healthcare systems, and professional identity. The overall internship experience and designated projects invite students to achieve the PBCs in a variety of ways, including collaborating with community partners, translating complex scientific information for different audiences, tailoring communication for stakeholders to promote psychosocial adaptation, facilitating the accessibility of information, and demonstrating knowledge of how GCs engage in and contribute to research. Furthermore, by collaborating directly with the leadership of PAOs, students gain familiarity with organizations that operate at the interface of science, clinical care, and advocacy.

**TABLE 1:** The Genetic Counseling Student Exchange Program's advocacy internship learning objectives.

	Objective 1	Objective 2	Objective 3	Objective 4
Internship Objective	Apply science writing and communication skills to create educational videos and write articles for families affected by rare disease	Learn how to be an effective advocate for patients and families affected by a particular condition (and the rare disease community, more broadly)	Employ the principles of health literacy in the creation of resources for a lay audience to ensure that the information is engaging and digestible	Aid families and patients in understanding their potential role and opportunities for involvement in research
Corresponding ACGC PBCs	1. Apply knowledge of genetics and genomics principles, genetic conditions, and testing technologies to the practice of genetic counseling	7. Embody the values of the genetic counseling profession	4. Communicate genetics and genomics information to clients, colleagues, and other community partners	4. Communicate genetics and genomics information to clients, colleagues, and other community partners
	4. Communicate genetics and genomics information to clients, colleagues, and other community partners	7b. Follow applicable professional ethical codes	4a. Tailor communication to specific individuals and audiences	5c. Demonstrate knowledge of how genetic counselors engage and contribute to the research process
	4b. Use a variety of approaches to communicate genetic and genomic information	6d. Collaborate with members of the care team, clients, and other community partner	4b. Use a variety of approaches to communicate genetics and genomic information	

*Note:* Below each objective are the Practice Based Competencies (PBC), abstracted directly from the Accreditation Council for Genetic Counseling (ACGC 2023a), which thematically correspond with the described internship learning goal.

### 4.4 Feedback and Iterative Improvement

As a part of our programmatic effort to evaluate and improve the GCSX advocacy internship, we maintain a record of completed internship projects and solicit informal feedback via REDCap forms from both students and participating PAOs. The PAO leaders evaluate the student's performance, describing areas of strength and areas for growth, and provide overall impressions and an assessment of the student's internship. Similarly, students provide feedback about the rotation experience, including what they liked best and what was least rewarding. Students also share suggestions for improvement for future internships. Students receive feedback from PAO evaluation forms and are prompted to meet with PAO leadership to review the feedback and close out the internship experience. These evaluations are identifiable and are reviewed by LJK and DR to inform program development, which introduces power imbalances that may impact what students and organizations feel comfortable sharing.

Although a thorough evaluation of the program's impact on learners, the PAOs, and program staff is beyond the scope of this paper, interim feedback has been vital for learning what works and what requires adaptation. For example, we initially launched the program as a 5‐week internship but extended it to 10 weeks as feedback from multiple students and PAOs expressed the desire for a longer experience to build relationships with each other. The longer internship also allowed time to collaborate on a project proposal to ensure project deliverables were feasible and aligned with PAO needs. Additionally, the initial launch of GCSX partnered multiple students to work with a single PAO; however, as interest and enthusiasm from participating PAOs expanded, the program has shifted to pairing a single student with each PAO. This appears to afford students greater ownership of their work while maintaining the interest of PAOs by ensuring a larger proportion of PAO applications are successfully matched with learners.

## 5 Reflections on the GCSX Advocacy Internship Experience

Across 4 years of GCSX advocacy internships, 72 students have collaborated with over 50 PAOs representing a wide variety of conditions. To give greater insight into the variability of the advocacy internship, Table 2 shows select collaborating PAOs, a summary of the associated intern projects, the practice‐based competencies developed via project completion, and illustrative quotes from both students and PAOs that we collected via written informal evaluations. Examples in Table 2 were chosen to highlight evaluations that provided the most robust feedback, although they are likely not representative of all internship experiences. Additionally, we chose examples to highlight the wide range of project deliverables and formats that PAOs solicited and that students were able to complete within the timeline.

**TABLE 2:** Select collaborating patient advocacy organizations (PAOs) are listed in the far‐left column followed by exemplary student internship projects.

Collaborating PAO	Project description	PBCs exemplified by project	Illustrative feedback from PAOs and GC students from informal evaluations
CureMito Foundation Supports patients and families impacted by Leigh syndrome and other mitochondrial diseases	A 6‐part “toolkit” for newly diagnosed Leigh Syndrome families, providing a step‐by‐step checklist for navigating the emotions of a diagnosis, maintaining parental well‐being, preparing for doctor appointments, and sharing news with others A family planning guide, a patient‐friendly resource explaining inheritance patterns for mitochondrial disease and reproductive options	1a: Described inheritance patterns for mitochondrial disease and reproductive planning options 3c: Provided tools and guides for a newly diagnosed family's psychosocial adaptation 4a: Tailored communication to be understood by patient families	“[The student] did an excellent job explaining very complicated information in a patient‐focused way, structuring the document in a way that's easy to follow, and formatting it, and now we have this guide available to help families, which is so needed.” – PAO leader, CureMito Foundation
National Fragile X Foundation Supports the Fragile X Syndrome community	A four‐part informational flier series covering the genetic concepts related to Fragile X, the associated phenotypes with premutation status, the genetic testing options for Fragile X, and the role of genetic counseling in clinical care	1b: Mobilized knowledge of Fragile X to present relevant information 4a: Presented information in a patient‐friendly way	“She also brought a background of knowledge that is helping our organization to provide additional information to our community.” – PAO leader, National Fragile X Foundation
APBD Research Foundation Dedicated to finding treatments for Adult Polyglucosan Body Disease and other forms of Glycogen Storage Disease IV while improving the lives of patients and families impacted	A “Who's Who?” document, synthesizing patient and caregiver information and contact information in a resource to share amongst the community to facilitate building connections within the group Assisted in reading a patient narrative in a live focus group webinar for Glycogen Storage Disease IV. The family did not speak English, so the student facilitated sharing their translated story in the meeting.	3c: Helped to promote psychosocial adaptation by helping to facilitate community‐building and development of a support network 7c: By sharing a translated narrative, aided in overcoming language barriers to ensure that diverse perspectives could be included in a condition‐specific meeting	“I enjoyed working with the APBD Research Foundation and getting to know a little bit about their community. It gave me a lot of insight on what it is like working in the rare disease space. I particularly enjoyed being involved in the Focus Group meeting; it was really wonderful hearing patient and family perspectives.” – GC Student
Schinzel‐Giedion Syndrome Foundation Supports families impacted by Schinzel‐Giedion Syndrome, a multisystemic genetic disorder caused by variants in *SETBP1*	A three‐part, patient‐friendly video series describing available clinical research enrollment opportunities for the patient community, including description of the patient registry, biorepository, and a urinary tract anomalies research study	4a: Tailored communication to be understood by patient families 5c: Demonstrated knowledge of how genetic counselors engage and contribute to the research process	“I learned more about clinical research as well as how to effectively communicate complex topics both in writing and visually… I really appreciated the collaborative nature of this internship and getting to participate in such meaningful work. I think this is a unique and important opportunity/experience for all future students.” – GC Student
Project CASK Supports individuals impacted by *CASK*‐related conditions, which encompass two main phenotypes: microcephaly with pontine and cerebellar hypoplasia (MICPHCH) and X‐linked intellectual disability	A written guide explaining genetic inheritance patterns and types of *CASK* variants, written both in English and in Portuguese A written guide providing information for families to interpret their child's genetic test report, written both in English and Portuguese	1a: Described genetic principles like inheritance patterns and types of variants 7c: By translating the work into another language, ensured the genetic information was accessible to an otherwise underserved population, promoting principles of equity and inclusion	“Truly appreciate the opportunity to provide educational resources to the CASK community especially when I was able to provide material in Portuguese.” – GC Student
Rory Belle Foundation Dedicated to supporting families affected by *NARS1* disorder, driving research, raising awareness, and fostering community	A comprehensive curation of published clinical and functional literature alongside patient‐reported RARE‐X data A presentation communicating to families the resulting knowledge and evidence gaps from the literature review to illustrate the value of their RARE‐X contributions and to gather input on their research priorities	1. Applied knowledge of the genetic condition and basic genomic principles to the developed materials 4b. Used multiple formats to communicate information 5c. Engaged in background research on the condition to facilitate the community's knowledge of current research	“I loved working with [PAO leader] and witnessing the passion she draws from Rory's story. She is a remarkable advocate, bringing the NARS1 community together while driving research forward, and I know she will continue to make impressive progress.” – GC Student

*Note:* The PBCs that correspond with each project are listed in the third column. Illustrative quotes abstracted from informal feedback from foundation leadership and genetic counseling trainees are highlighted in the final column.

### 5.1 Student Experience

Informal feedback from evaluative forms, internship projects, and class and advising conversations with students suggests that the GCSX advocacy internship is a positive educational experience. The process of developing patient‐friendly educational resources or other community‐tailored deliverables encourages many learners to practice and hone effective communication skills relevant for genetic counseling practice and explicitly outlined in ACGC PBCs. Additionally, in having learners collaborate to contribute to the materials a PAO offers to their patient community, the internship provides what MK describes as a “look behind the curtain of what exactly an advocacy group does,” acquainting students with the types of resources, efforts, and future directions that groups prioritize for patient communities.

While some students' experiences are strictly focused on developing educational materials, for others, informal reflections demonstrate that the internship acquaints them with the lived experience of rare disease and disability because many of the participating organizations students work with are run by the parents of children with rare genetic diagnoses. In meeting regularly with PAO leadership and attending community meetings to gain insights and feedback on their projects, some hear about traversing diagnostic odysseys, navigating complex informational needs, and the day‐to‐day challenges of a rare disease diagnosis. One student told us about feeling the race against the clock that parents experience as they push for novel treatments, while another described beginning to grasp the weight of uncertainty on parents, given how little is often known about the natural history of rare conditions, let alone the social course of their children's futures in adulthood. Another student described hearing directly from patient families, who described the challenges encountered in navigating disability support services in school systems.

Additionally, as some learners' projects are centered around PAO's goals to advance disease‐specific research, the experience provides an appreciation for the relevance and centrality of the patient community voice in advancing therapeutic development. During their advocacy internships, some students describe a growing understanding of the breadth and complexity of a community‐driven research agenda as parents involved in PAOs have prioritized concerns about diagnostic delays and the impact of specific rare diagnoses on sleep, attention, and anxiety, in addition to a search for cures. Finally, through participating in the advocacy experience, students articulate a deeper awareness of the role of advocacy in genetic counseling professional identity. As one student shared, the internship “showed me that advocacy is not separate from genetic counseling, but a key part of the role.”

### 5.2 PAO Experience

The PAOs who have participated in the GCSX are varied. Many of the participating PAOs are gene‐specific or condition‐specific organizations, while others, like the Rare Epilepsy Network, are umbrella groups that encompasses hundreds of genetic diagnoses. From pediatric to adult conditions, and ultra‐rare to more common rare conditions, participating PAOs represent a wide range of individuals and families. They also include PAOs with international reach, such as Fundación Duchenne Chile, enabling students with bilingual or bicultural backgrounds or interests to gain experience working with PAOs working beyond U.S. borders.

Despite the variability in PAO characteristics, evaluative feedback from PAOs generally conveys that organizations perceive student work to be useful to their communities. One organization commented soon after the internship that the student's project “has been beneficial to our community already,” while another PAO shared feeling “grateful” for the resources that the student developed, “which will be very helpful for [families] as they learn the diagnosis for their child.” Indeed, many GCSX projects focus on developing durable resources for PAOs to share with the families that they serve. The lack of rigorous research on outcomes of the GCSX inhibits our ability to generalize PAO's perspectives on their experience across internships; however, the fact that many participating groups apply to participate in multiple cycles of the GCSX advocacy internship suggests, though does not prove, that learner involvement is useful to them. For example, the Rare Epilepsy Network (REN) worked with DR on the concept of advocacy projects and proposed ideas for projects that would benefit REN's members. This included development and promotion of a Genetic Testing Toolkit that is readily accessible on the organization's website. REN was an early and repeat participant in GCSX and promotes GCSX to its member groups, many of which have also participated.

This enthusiastic engagement from participating PAOs is also evidenced by ongoing collaboration extending beyond the formal rotation on several occasions, including research assistance, thesis project proposals, collaboration to host a rotation for an undergraduate internship program in genetic counseling, and a rotation for genetic counseling assistants at Penn and our associated children's hospital, CHOP.

### 5.3 JumpStart Experience

The Genetic Counseling Student Exchange (GCSX) Program, a joint initiative of the Orphan Disease Center's JumpStart Program at Penn and Penn's MSGC Program, has emerged as a powerful mechanism to bridge academic training and patient‐driven research. The JumpStart program was designed to connect PAOs, researchers, and key opinion leaders, often supporting organizations that lack the infrastructure or expertise to independently drive research forward. Within this framework, the GCSX program provides an efficient and impactful extension of support from JumpStart. For JumpStart partners, many of which operate with limited scientific or communication resources, this model provides critical, high‐quality support that advances their outreach goals while strengthening connections to the academic community.

PAOs consistently demonstrate strong interest in hosting students, and the program has experienced growing demand. Each year, more organizations express interest in hosting students and propose well‐defined, high‐impact projects—often exceeding the number of available trainees. This highlights both the demand for scientific communication support and the program's growing reputation. The rising engagement underscores both the unmet needs within the rare disease ecosystem and the mutual benefit of these collaborations: students have real‐world interactions with rare disease communities, while PAOs expand their reach, better engage patient communities, and more effectively communicate research progress, all of which are critical for advancing therapeutic development.

Given its scalability, relatively low cost, and demonstrated impact, expanding this model to additional master's‐level genetic counseling programs represents a strategic opportunity for our programs. Broadening participation would not only meet increasing demand from PAOs but also cultivate a workforce of genetic counselors trained in advocacy, research translation, and community engagement—skills that are essential to accelerating progress across the rare disease landscape. Of note, the GCSX program has expanded beyond Penn's genetic counseling program to include students from Vanderbilt University, the University of Pittsburgh, Rutgers University, Stanford University, and the Massachusetts General Hospital Institute of Health Professions. Students have organized these experiences with the JumpStart director who matched them with national PAOs following the structure and format developed with the Penn program. Though these internships are one‐off experiences initiated by students at other genetic counseling graduate programs, genetic counseling programs may be able to leverage their GC alumni networks, our GCSX materials, and connections through JumpStart to consider developing similar advocacy internships at their universities.

### 5.4 Penn MSGC Program Leadership Experience

By sequencing the advocacy internship relatively early in our students' clinical education, we find that the internship is especially poised to facilitate students' development of a professional identity that aligns with a core competency outlined in Section 7 of the PBCs (Accreditation Council for Genetic Counseling 2023a). Professional identity formation is a complex construct involving socialization into the profession and a sense of belonging (Cornett et al. 2023). Contributing genetics expertise to a PAO through the internship reinforces students' awareness of their developing genetic expertise and their role in the genetic counseling profession. Completing the advocacy internship prior to their first active clinical rotation provides students with a deeper understanding of the lived experiences of patients, such as access and barriers to genetic counseling and testing, and strategies for patient advocacy outside of the clinical space.

By working on behalf of rare communities rather than with individual patients, the advocacy internship is also particularly helpful for teaching students about Section 4 of the NSGC Counselor's Code of Ethics, which stipulates GC's relationship and responsibilities related to society more generally (National Society of Genetic Counselors 2017). In teaching the Code of Ethics to GC students, we have realized they need more exposure and guidance on what it means to educate the public and to promote policies that advance ethically responsible research. As a part of the GCSX advocacy internship, students often exercise the skill of translating complex information for different communities, including parents, schools, or fundraisers, which begins to equip them to play a role in educating varied stakeholders about diagnostics and rare conditions.

While the GCSX advocacy internship program has begun to evolve and expand beyond Penn, our view of its central goals remains unchanged: to expose students to rare disease communities and patient advocacy efforts, to develop their skills relevant to the genetic counseling profession, and to educate them about community priorities and perspectives via a service‐learning model.

## 6 Reflections on Advocacy Training as Disability Education in Genetic Counseling

In implementing an experiential learning program through the GCSX advocacy internship, we recognize that a handful of early genetic counseling training programs had also championed disability advocacy through community‐based experiences (Hogan 2022; Stern 2012). With our advocacy internship program, we are therefore beginning to come full circle, as we aspire to formalize students' exposure to, and assistance with, advocacy. While early efforts to place disability advocacy at the forefront of genetic counseling programs generally focused on equipping students with knowledge of disability to incorporate into clinical counseling (Hogan 2022), in the current era, the advocacy internship also aims to prepare students to work within advocacy organizations. This is especially timely given the increasingly large role PAOs play in pushing precision medicine forward (Koay and Sharp 2013; Merkel et al. 2016; Patterson et al. 2023). Furthermore, it is also timely given the recent recognition that the standard for disability education in genetic counseling programs is all too limited (Douglas et al. 2023; Houtz and Mueller 2025; Miller et al. 2025; Sanborn and Patterson 2014; Winchester et al. 2024).

Amid calls for more robust disability education within genetic counseling, we see the advocacy internship as a step in developing targeted disability education for genetic counseling trainees. Given that many of our partner organizations focus on conditions associated with disabilities, we hope that the advocacy internship experiences can be leveraged to increase students' awareness of the lived experience of disability. Although often the advocacy internship focuses on what students can do for their PAOs, PAO leadership and community volunteers play a vital role in helping students gain exposure to families' lived experiences of genetic conditions by identifying what types of resources must be collated to equip parents and patients to navigate life with rare disease. Depending on the projects organizations prioritize, some students gain an appreciation for the challenges of navigating state support for children with disabilities or the resources desired by a patient community to better navigate the social aspects of disability, such as individualized education plans. The importance of exposure to the lived experience of disability has been identified by disability advocates as an effective method for educating genetic counseling trainees on disability topics (Winchester et al. 2024), suggesting that the GCSX may be an important step in teaching GC students about disability.

By exposing students to condition‐specific communities beyond clinical contexts, the advocacy internship may increase some learners' understanding of social factors, such as social service policies, that impact experiences of illness and disability, while better acquainting them with available resources and supports, which several students have been charged with reviewing, editing, and organizing (Douglas et al. 2023; Winchester et al. 2024). Through their advocacy rotations, some students also learn in‐group lingo like “8p heroes” or “Syngaptians,” terms that rare communities use to celebrate, rather than pathologize, their people. These informative, memorable interactions are not part of the advocacy curriculum; they are what can happen organically when we require our students to commit time and energy to connecting with and serving advocacy organizations.

While the advocacy internship aims to start heeding the call for better disability education, we recognize the limitations of experiential engagement without a formalized didactic curriculum focused on disability. This is especially critical because the GCSX advocacy internship is a heterogeneous experience given the variability of PAOs and prioritized projects. While some students share the many insights that working with parents of affected children has given them, others describe less informative experiences that were focused mainly on developing genetic testing or educational resources, absent robust, recurrent, and personal interactions with PAO leadership or community. Increasingly, we see a need to augment the advocacy internship with group reflection, intentional activities, and tailored lectures on disability in genetic counseling, medicine, and society more generally. This would enable students to compare their experiences across different internship sites and learn from the breadth of participating PAOs. These group reflections could be paired with canonical lessons from the disability rights movement, adult advocates with disabilities, and disability scholarship that typically gets insufficient attention in GC graduate education. For example, teaching genetic counseling students to discern different models of disability (Shuman 2024) and to apply them within the context of their advocacy internships is critical because many advocacy organizations are founded and run by parents of affected children who often primarily subscribe to a medical model of disability, rather than the social model that is often favored by disability self‐advocates (Houtz and Mueller 2025). Additionally, though the ten‐week timeline limits student involvement in policy change, adding case examples of instances when PAOs have individually or collectively prompted key shifts in policy could ensure that additional didactics contextualize the broader efforts many PAOs engage in, such as advocating for passage of the Orphan Drug Act and the Food and Drug Administration's (FDA) Patient‐Focused Drug Development.

Indeed, given FDA's increased focus on patient‐centered outcome measures and the role of PAOs in the development of rare therapeutics, the advocacy internship has the potential to expose students to infrastructure and strategies for ensuring that patient and caretaker perspectives are incorporated into clinical research and the development of novel therapies (Berg et al. 2024). Including instruction that gives students a vocabulary and framework for organizing their experiences and observations could make these lessons explicit and generalizable. By collaborating with a growing roster of PAOs, the advocacy internship provides an opportunity to expose genetic counseling students to an ecosystem in which patient organizations are not peripheral voices but co‐creators of knowledge, therapies, and policy. Thus, in linking academic training with patient‐driven initiatives, the experience aims to educate genetic counseling trainees about how to advance socially responsive rare disease research and care.

By highlighting the feasibility of the advocacy internship for students at Arcadia, Penn, and several other MSGC programs, we hope that this paper provides justification, an example framework, and additional ideas for genetic counseling programs to consider implementing advocacy experiences into their curricula. By explicitly teaching students how genetic counseling skills can be applied in advocacy settings, we also hope to begin to foster professional identities that are inclusive of diverse roles in varied practice settings given how the field is evolving and expanding. Furthermore, by placing the priorities of each PAO at the center of the advocacy internship, we hope to equip genetic counseling trainees with the skills and values to learn about and champion the needs of people living with genetic conditions and disabilities.

## 7 Conclusion and Future Directions

While the GCSX internship appears popular with students, graduate program applicants, and PAOs, our knowledge of learner and PAO experiences with the internship program is limited to REDCap evaluations and various informal reflections shared in meeting, advising, and coursework conversations. Given this and the heterogeneity of organizations and internship projects, it will be important to formally evaluate GCSX via a mixed methods approach, ideally as a genetic counseling master's thesis. Evaluating the motivations, experiences, and evolving needs of participating PAOs is key to understanding the program's impact on organizations and communities. Assessing the experiences of PAOs and learners, as well as of program leadership who assist with the internship and graduate education, is important for evaluating the existing programming and associated time demand on MSGC and PAO staff, and for identifying ways to strengthen the internship program. A mixed methods approach is important to quantify achievement of different learning objectives from the perspectives of learners and PAOs, while also performing exploratory research to better understand what casual interactions with PAO leadership and community members teach students and how these interactions can be cultivated, if they are confirmed to be valuable. Rigorous evaluation of the experiences of PAOs with GCSX will also be critical to ensure that the program consistently benefits organizations and communities and instills what they deem most important for GCSX learners.

If the advocacy internship is confirmed to offer insight into the lived experience of disability, we plan to design a disability curriculum around it. To do this, we would enlist disability and rare disease advocates who have both caretaking and individual experience of different disabilities to identify key concepts and diverse perspectives on rare disease and disability to inform the curriculum. DisabilityGC, an informal working group of GCs with disabilities, could also be consulted to help identify additional concepts, perspectives, and individual and group reflective practices to round out a disability advocacy curriculum. This collaborative and consultative approach could help to frame and address the over‐representation of parent‐run organizations focused on medical innovation among available internship sites. Via inclusion of lectures by disability self‐advocates, we hope to build instruction that both clarifies differences in perspectives and introduces students to organizations run by people living with different conditions such as Little People of America or Autism Self‐Advocacy Network.

For MSGC programs that may be considering integrating advocacy rotations or experiences into their fieldwork offerings, we recommend leveraging existing networks to identify alumni and other connections such as program lecturers, conference speakers, and genetic counseling supervisors and colleagues who may be personally or professionally involved with PAOs and who could be approached to host advocacy internships. We emphasize starting small, as we did with one and then two alumni connections, to place just a few students to see what works within the confines of a given program. In approaching PAOs that may potentially host advocacy internships, we advise learning about the organizations' current needs, such as conference planning or volunteers, to propose internship experiences that align learner objectives with PAO priorities so that gains are reciprocal and goals are synergistic. Based on our experience iterating the GCSX program and our remaining questions about its overall impact, we also recommend incorporating outcome measures that are both descriptive and quantitative so that the field can learn how to most effectively teach students about advocacy and the lived experiences of rare disease and disability.

## Author Contributions

Marin Kalista, Lisa Jay Kessler, Deborah Requesens, and Rebecca Mueller were responsible for conceptualization, preparing and writing the original draft, and reviewing and editing the manuscript. Lisa Jay Kessler, Deborah Requesens, and Kathleen D. Valverde were responsible for project administration. Kathleen D. Valverde reviewed and edited the manuscript.

## Funding

This work was supported by The Warren Alpert Foundation, Alliance for Genetic Counseling (PD 10084768‐03) and National Human Genome Research Institute (1K01HG013339).

## Disclosure

Artificial Intelligence: Artificial Intelligence was not used to draft this manuscript.

## Ethics Statement

Since this is an educational program and not human subjects research, no IRB approval was necessary.

## Conflicts of Interest

The authors declare no conflicts of interest.

## Data Availability

There is no data related to this manuscript beyond student internship records. Permission to quote such records was granted by students and patient advocacy organizations.
